# 培美曲赛单药与培美曲赛联合奥沙利铂用于Ⅳ期肺腺癌挽救性治疗的比较

**DOI:** 10.3779/j.issn.1009-3419.2011.09.01

**Published:** 2011-09-20

**Authors:** 友如 刘, 银铃 江, 志强 高, 向迎 王, 宝惠 韩, 丽岩 姜

**Affiliations:** 200030 上海，上海交通大学附属胸科医院肺内科 Department of Pulmonary, Chest Hospital Affiliated to Shanghai Jiaotong University, Shanghai 200030, China

**Keywords:** 肺肿瘤, 培美曲赛, 奥沙利铂, 吉非替尼, 厄洛替尼, Lung neoplasms, Pemetrexed, Oxaliplatin, Gefitinib, Erlotinib

## Abstract

**背景与目的:**

目前肺癌挽救性治疗尚无标准方案。本研究旨在比较培美曲赛单药与培美曲赛联合奥沙利铂挽救性治疗Ⅳ期肺腺癌患者的疗效及安全性，为联合化疗提供依据。

**方法:**

2009年1月-2011年2月共83例体能状态评分（performance status, PS）为0分-2分的Ⅳ期肺腺癌患者分别接受培美曲赛（单药组47例）和培美曲赛联合奥沙利铂（联合组36例）挽救性治疗，观察两组近期疗效和毒性反应并进行比较。

**结果:**

81例患者纳入最终分析。单药组与联合组中位无进展生存时间（progression-free survival, PFS）分别为3.6个月*vs* 4.1个月（*P*=0.268），客观反应率（objective response rate, ORR）和疾病控制率（disease control rate, DCR）分别为6.5% *vs* 20%（*P*=0.092）和56.5% *vs* 65.7%（*P*=0.493）。单药组与联合组血液毒性及胃肠道反应发生率分别为33.9% *vs* 47.2%（*P*=0.460）和21.2% *vs* 25.0%（*P*=0.213）。

**结论:**

培美曲赛联合奥沙利铂挽救性治疗PS评分较好的Ⅳ期肺腺癌患者耐受性良好，与培美曲赛单药相比显示出较高的缓解率，但未明显增加患者的PFS。

## Abstract (en)

**Background and objective:**

At present, there is no standard salvage treatment strategies for lung cancer. The aim of this study is to compare the efficacies and safeties of pemetrexed alone with pemetrexed combined with oxaliplatin as salvage therapy in stage Ⅳ lung adenocarcinoma to provide evidences for combination therapy.

**Methods:**

From January 2009 to February 2011, 83 patients with stage Ⅳ lung adenocarcinoma received pemetrexed alone (single agent arm, *n*=47) or pemetrexed combined with oxaliplatin (combination arm, *n*=36) as salvage therapy. All 83 patients had performance status (PS) scores of 0-2.

**Results:**

Eighty-one patients were included in the final analysis. The median progression-free survival (PFS) in the single agent arm was 3.6 months versus 4.1 months in the combination arm (*P*=0.268). The objective response rate (ORR) was 6.5% versus 20% (*P*=0.092), and the disease control rate (DCR) was 56.5% versus 65.7% (*P*=0.493), respectively. The response rates of the hematological and gastrointestinal toxicities in the single agent and combination arms were 33.9% versus 47.2% (*P*=0.460) and 21.2% versus 25.0% (*P*=0.213), respectively.

**Conclusion:**

For salvage therapy, pemetrexed combined with oxaliplatin is tolerable in stage Ⅳ lung adenocarcinoma patients with good PS scores. Compared with pemetrexed alone, pemetrexed combined with oxaliplatin therapy showed higher response rate, but did not significantly prolong the PFS.

肺癌是危害人类健康的主要恶性疾病之一，也是导致癌症死亡的首要原因^[1]^。其中非小细胞肺癌（non-small cell lung cancer, NSCLC）约占肺癌总数的80%，且多数患者确诊时病情已属晚期。目前晚期NSCLC的主要治疗方法为包括化疗和靶向药物在内的综合治疗。铂类联合第三代化疗药物的双药方案为晚期NSCLC的标准一线治疗方案^[2]^。在二线或三线治疗中小分子靶向药物表皮生长因子受体酪氨酸激酶抑制剂（epidermal growth factor receptor-tyrosine kinase inhibitors, EGFR-TKIs）吉非替尼（gefitinib）和厄洛替尼（erlotinib）已成为标准治疗之一，尤其对肺腺癌患者疗效较好^[3-5]^。然而大多数患者靶向治疗后病情进展或产生耐药，如何进一步治疗这部分人群成为当下研究的热点。

培美曲赛（pemetrexed）用于NSCLC的挽救性化疗，可使40%-50%的患者疾病得到短期控制^[6, 7]^。一项临床Ⅱ期研究^[8]^提示培美曲赛联合奥沙利铂（oxaliplatin）在经治的体能状态评分（Eastern Cooperative Oncology Group performance status scale, ECOG-PS）较好的晚期NSCLC患者中耐受良好，同时Scagliotti等^[9]^报道培美曲赛联合奥沙利铂较培美曲赛联合卡铂在晚期NSCLC患者中取得了较好疗效且安全性更好。对于靶向治疗后病情进展或耐药的患者，在PS评分较好的人群中培美曲赛联合奥沙利铂是否较培美曲赛单药能带来更多获益？为此，本研究回顾性分析2009年1月-2011年2月在上海交通大学附属胸科医院接受培美曲赛（力比泰）单药或培美曲赛（力比泰）联合奥沙利铂（乐沙定）挽救性治疗的Ⅳ期肺腺癌患者的病例资料，评价两种治疗方案的疗效及安全性，旨在为Ⅳ期肺腺癌患者的联合化疗提供依据。

## 1 材料与方法

### 1.1 研究对象

共83例PS评分为0分-2分的患者接受培美曲赛（单药组47例）和培美曲赛联合奥沙利铂（联合组36例）治疗。单药组接受培美曲赛500 mg/m^2^，第1天；联合组接受培美曲赛500 mg/m^2^，第1天，联合奥沙利铂120 mg/m^2^，第1天，每3-4周为1个周期，并给予叶酸、VitB12及地塞米松处理。所有患者均符合以下标准：①经组织或细胞病理学证实为肺腺癌；②既往接受含铂化疗和吉非替尼或厄洛替尼治疗且病情进展或耐药；③根据国际抗癌联盟（Union for International Cancer Control, UICC）第七版NSCLC TNM分期为Ⅳ期的肺腺癌患者；④挽救性治疗前血常规及肝、肾功能等指标基本正常，无严重心脏病和其它合并症；⑤既往未接受过培美曲赛化疗。两组患者的具体情况见表 1。

**1:** 81例挽救性治疗的Ⅳ期肺腺癌患者的临床特征 The clinical characteristics of 81 phase Ⅳ lung adenocarcinoma patients received salvage therapy

Variable	Single agent (*n*=46)	Combination (*n*=35)	*P*
Median age (range)	62 yr (40-77)	55 yr (26-77)	0.002
Age (yr)			0.025
≥60	28 (60.9%)	12 (34.3%)	
＜60	18 (39.1%)	23 (65.7%)	
Gender			0.173
Male	22 (47.8%)	11 (31.4%)	
Female	24 (52.2%)	24 (68.6%)	
Smoking history			0.098
Yes	13 (28.3%)	4 (11.4%)	
No	33 (71.7%)	31 (88.6%)	
ECOG PS score			0.725
0-1	40 (87.0%)	32 (91.4%)	
2	6 (13.0%)	3 (8.6%)	
Surgery history			0.790
Yes	11 (23.9%)	7 (20.0%)	
No	35 (76.1%)	28 (80.0%)	
Radiotherapy history			0.115
Yes	14 (30.4%)	5 (14.3%)	
No	32 (69.6%)	30 (85.7%)	
First-line chemotherapy history			0.999
Cisplatin-based chemotherapy	30 (65.2%)	23 (65.7%)	
Carboplatin-based chemotherapy	16 (34.8%)	12 (34.3%)	
Second-line chemotherapy history			0.999
Yes	14 (30.4%)	10 (28.6%)	
No	32 (69.6%)	25 (71.4%)	
Extra-thoracic metastasis			0.232
Yes	34 (73.9%)	21 (60.0%)	
No	12 (26.1%)	14 (40.0%)	
EGFR-TKIs treatment			0.493
Gefitinib	26 (56.5%)	23 (65.7%)	
Erlotinib	20 (43.5%)	12 (34.3%)	
ECOG-PS: Eastern Cooperative Oncology Group performance status scale.			

### 1.2 方法

末次随访时间为2011年4月30日，随访方式为电话随访，中位随访时间为7.4个月。主要研究终点为无进展生存时间（progression-free survival, PFS），即从第1次挽救治疗日期开始至客观证据表明疾病进展或末次随访时间。次要研究终点为客观反应率（objective response rate, ORR）、疾病控制率（disease control rate, DCR）和药物毒性反应。按照实体瘤疗效评价标准（Response Evaluation Criteria in Solid Tumors, RECIST 1.1）统一评价疗效，分为完全缓解（complete response, CR）、部分缓解（partial response, PR）、稳定（stable disease, SD）和进展（progressive disease, PD），其中ORR=CR+PR，DCR=CR+PR+SD。按NCI常见毒性分级标准（CTC 3.0版）评价毒性反应。

### 1.3 统计学方法

应用SPSS 13.0统计软件进行统计分析，年龄比较采用秩和检验，临床特征、疗效和毒性反应比较应用*Fisher*确切概率法。PFS采用*Kaplan-Meier*法进行分析。采用单因素分析及*Cox*模型进行预后分析。*P* < 0.05为差异有统计学意义。

## 2 结果

### 2.1 疗效反应

83例患者中2例（2.4%）失访，共81例纳入最后疗效分析，其中单药组46例，联合组35例。单药组和联合组中位PFS分别为3.6个月（95%CI: 2.10-5.10）和4.1个月（95%CI: 2.90-5.30），差异无统计学意义（χ^2^=1.23, *P*=0.268）。两组PFS生存曲线见图 1。纳入分析的81例患者均可评价疗效，两组无CR患者，单药组和联合组分别有3例（6.5%）和7例（20%）达到PR（*P*=0.092），单药组和联合组患者的DCR分别为56.5%（26例）*vs* 65.7%（23例）（*P*=0.493）。

**1:**
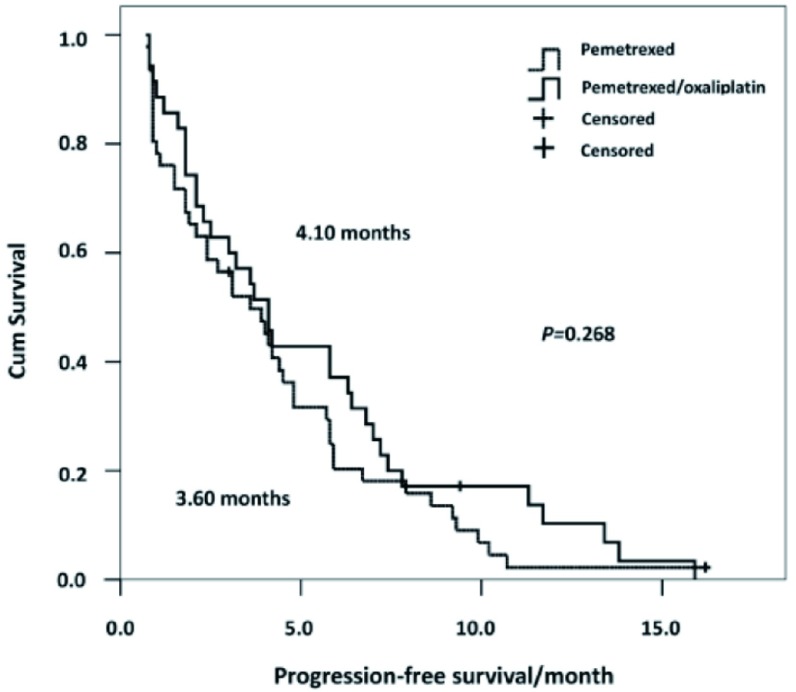
81例挽救性治疗的Ⅳ期肺腺癌患者的无进展生存曲线 Progression-free sur vival cur ves of 81 stage Ⅳ lung adenocarcinoma patients receiving salvage therapy

### 2.2 毒性反应

83例患者均可评价药物毒性反应（表 2）。主要毒性反应为血液毒性和胃肠道反应，单药组与联合组发生率分别为33.9% *vs* 47.2%（*P*=0.460）及21.2% *vs* 25.0%（*P*=0.213）。其它毒性包括肝功能异常、皮疹、疲乏、外周神经毒性等。单药组1例因Ⅳ度骨髓抑制、粒缺性发热导致治疗中断；联合组因1例Ⅲ度骨髓抑制并肝功能异常及1例Ⅲ度胃肠道反应而停用奥沙利铂。

**2:** 83例挽救性治疗的Ⅳ期肺腺癌患者的毒性反应 The toxic side effects of 83 phase Ⅳ lung adenocarcinoma patients received salvage therapy

Toxic side effects	Single agent (*n*=47)					Combination (*n*=36)				*P*
	Ⅰ	Ⅱ	Ⅲ	Ⅳ		Ⅰ	Ⅱ	Ⅲ	Ⅳ	
Hematological toxicity	9	5	1	1		8	6	3	0	0.460
Gastrointestinal toxicity	5	5	0	0		7	1	1	0	0.213
Abnormal liver function	1	0	0	0		2	1	0	0	0.391
Fatigue	4	1	0	0		4	0	0	0	0.845
Rash	2	1	0	0		2	0	0	0	0.999
Peripheral neurotoxicity	0	0	0	0		2	1	0	0	0.078
Fever with neutrophils	0	0	1	0		0	0	0	0	0.999

### 2.3 预后因素分析

在以培美曲赛为基础的挽救性治疗中，单因素分析显示治疗前PS评分（HR=6.310, 95%CI:2.806-14.191, *P* < 0.001）和既往化疗及靶向治疗疗效（HR=1.645, 95%CI:1.021-2.650, *P*=0.041）对患者的PFS有明显影响，既往手术治疗史（HR=0.601, 95%CI: 0.346-1.044, *P*=0.071）具有影响PFS的趋势。多因素分析显示治疗前PS评分（HR=6.310, *P* < 0.001）和既往化疗及靶向治疗疗效（HR=2.297, *P*=0.049）是肺腺癌患者挽救性治疗PFS的独立影响因素，既往手术治疗史（HR=0.540, *P*=0.059）具有影响PFS的趋势。具体结果见表 3。

**3:** 81例挽救性治疗的Ⅳ期肺腺癌患者PFS单因素及多因素分析 Single-factor and multivariate analysis of PFS for 81 phase Ⅳ lung adenocarcinoma patients receiving salvage therapy

Variable	*n*	PFS (month)	Single-factor analysis				Multivariate analysis	
			HR	95%CI	*P*		HR	*P*
Gender			0.909	0.569-1.451	0.690		0.906	0.790
Male	33	3.6						
Female	48	4.1						
Age (yr)			0.836	0.535-1.392	0.545		0.933	0.806
≥60	40	4.1						
＜60	41	3.6						
Smoking history			0.775	0.444-1.354	0.371		0.912	0.816
Yes	17	2.7						
No	64	4.1						
ECOG PS score			6.310	2.806-14.191	< 0.001		6.31	< 0.001
0-1	72	4.1						
2	9	1.0						
Surgery history			0.601	0.346-1.044	0.071		0.540	0.059
Yes	18	5.7						
No	63	2.7						
Radiotherapy history			1.590	0.904-2.799	0.108		1.449	0.245
Yes	19	4.4						
No	62	3.2						
Second-line chemotherapy history			0.695	0.411-1.174	0.173		0.689	0.270
Yes	24	3.0						
No	57	4.0						
Extra-thoracic metastasis			1.202	0.734-1.970	0.465		1.023	0.940
Yes	55	3.6						
No	26	4.2						
EGFR-TKIs treatment			1.081	0.635-1.630	0.942		1.314	0.448
Gefitinib	49	4.1						
Erlotinib	32	3.1						
PFS with EGFR-TKIs			1.369	0.865-2.165	0.180		1.862	0.150
≥6 months	36	4.1						
＜6 months	45	3.1						
Disease control after both prior therapies			1.645	1.021-2.650	0.041		2.297	0.049
Yes	32	4.2						
No	49	2.7						
PFS: progression-free survival; EGFR-TKIs: epidermal growth factor receptor-tyrosine kinase inhibitors.								

## 3 讨论

培美曲赛是多靶点的叶酸拮抗剂，通过干扰细胞复制过程中叶酸代谢途径而发挥抗肿瘤作用。大型随机Ⅲ期临床研究^[10, 11]^已证实培美曲赛在一线、二线化疗中疗效不劣于吉西他滨和多西他赛，在肺腺癌患者中效果更好，毒副反应明显降低。本研究中单药组患者取得了令人满意的治疗效果，中位PFS为3.6个月，比Wu等^[6]^在文献中报道的3.0个月略长，可能与本研究的病例的PS评分较好有关。本研究中联合组取得了20%的缓解率，明显高于单药组的6.5%，结果略好于2009年荟萃分析^[12]^中晚期NSCLC二线双药化疗15.1% *vs*单药7.3%的总体缓解率。此结果可能与本研究中联合组合用奥沙利铂有关。既往文献^[13]^表明奥沙利铂作为第三代铂类药物，与顺铂、卡铂具有不完全交叉耐药性，适用于既往接受顺铂、卡铂治疗敏感或耐药患者。本研究中联合组中位PFS为4.1个月，略长于单药组3.6个月，但未达到统计学意义，与荟萃分析^[12]^中腺癌亚组双药PFS明显长于单药不同，结果的差异可能与本研究样本量较小有关。尽管总体PFS未达到统计学意义，但发现治疗中PR患者PFS明显延长，10例PR患者的中位PFS达10.2个月（95%CI: 3.2-17.2），而38例SD患者的中位PFS为5.7个月（95%CI: 4.4-7.0）。联合奥沙利铂有利于提高挽救性治疗人群PR患者比例，为PR患者带来更大临床获益。

本研究中单药组年龄≥60岁的患者比例高于联合组（*P*=0.025）。进一步的分析结果显示单药组与联合组年龄≥60岁和年龄 < 60岁患者中位PFS分别为4.0个月*vs* 4.1个月（*P*=0.920）及2.4个月*vs* 3.6个月（*P*=0.512），年龄因素不足以对两组PFS产生明显影响。与既往研究^[7]^相似，本研究亦得出PS评分是挽救性化疗患者PFS的独立影响因素：PS评分为0分-1分的患者PFS较PS评分为2分的患者明显延长（*P* < 0.001）。然而本研究中PS评分为2分的患者亚组病例数只有9例，可能会对预后分析结果产生一定影响。本研究中既往化疗和靶向治疗疾病均得到控制的患者PFS较其他患者延长（*P*=0.049），与Girard等^[14]^报道相吻合，提示既往化疗和靶向治疗疗效均较好的患者在靶向治疗进展后更应积极接受挽救性治疗。另外，单因素分析及*Cox*模型均提示手术治疗史具有影响PFS的趋势。手术治疗减轻了肿瘤负荷，肿瘤异质性低于初诊的进展期肺癌，因此在挽救性治疗中疾病相对容易得到控制。既往文献^[6, 7]^报道靶向治疗PFS≥6个月的患者更容易从挽救性治疗中获益。本研究中两类患者之间的PFS未出现明显差异，分析可能与本研究中联合组对靶向治疗PFS < 6个月的患者疗效相对优于单药组有关（中位PFS为3.7个月*vs* 2.4个月），而对于靶向治疗PFS≥6个月的患者，两组中位PFS均为4.1个月。

本研究中观察到的毒副反应轻于既往报道^[9, 13]^，联合组与单药组相比未见明显差异，两组患者耐受性良好，全组仅3例患者被迫中断治疗或停药。单药组76.2%（32/42）和联合组78.8%（26/33）的进展患者接受了进一步的化疗或/和靶向治疗。

本研究为回顾性分析，由于病例入选条件的限制以及研究样本量相对偏小，结果的可信度有待进一步提高。另外，由于本研究入选患者大多由细胞病理学明确诊断，且部分术后复发患者亦未常规进行组织标本EGFR表达或突变的检测，分子水平的差异是否会影响治疗疗效还需在今后的研究中加以论证。

总之，培美曲赛联合奥沙利铂用于PS评分较好的Ⅳ期肺腺癌患者挽救性治疗耐受性良好，与培美曲赛单药相比显示出较高的缓解率，但未明显增加患者的无进展生存时间。两组的远期疗效有待进一步观察。
